# Supplementary material for: General Public’s Knowledge of Diabetes and Physical Activity in Saudi Arabia over Time: The Need to Refresh Awareness Campaigns
Source: Healthcare (Basel). 2023 Jan 17;11(3):286. doi: 10.3390/healthcare11030286 (PMC9913913; doi:10.3390/healthcare11030286)
Supplement: Supplementary file 1 [file healthcare-11-00286-s001.zip › healthcare-2171687-supplementary.pdf]

Supplementary Table S1: Questionnaire

Diabetes is a widespread health problem, and millions of people around the world live with it, and it is increasing day by day. Please fill out the following questionnaire to find out what you know about diabetes:

1. Age: □Less than 15 years old □15-25 years old □26-35 years old □36-45 years old □46-55 years old 56-65 years old More than 65 years old
2. Gender: □ Female □Male
3. Marital Status: □ Single □ Married □ Widow □ Divorced
   Educational level: □ no official education □ diploma and below □ bachelor □postgraduate
4. Date: / /
5. Do you have a relative living with Diabetes? □Yes □ No
6. Nationality:

**No.**	**DM Knowledge and Awareness**	**Yes**	**No**	**I don't know**
**1.**	Obesity causes DM			
**2.**	Full or partial shortage of insulin hormone in the blood causes DM			
**3.**	Type 1 DM is a chronic disease with no available cure the time being, but it can be managed.			
**4.**	In healthy individuals, fasting blood sugar levels are considered high if above 100 mg/dcl.			
**5.**	Eyes functions could be affected by chronic high blood sugar levels and may lead to blindness.			
**6.**	Uncontrolled type 1 DM patient should not fast.			
**7.**	Polyuria (frequent urination) during night, sudden/ unexplained weight loss and excessive thirst are symptoms of hyperglycemia.			
**8.**	Gestational diabetes mellitus may increase the risk of giving birth to an overweight baby and might lead to her/his death.			
**9.**	Type 2 DM is a chronic disease with no available cure the time being, but it can be managed.			
**10.**	The prevalence of DM is high in the community and it increased in recent years.			
**11.**	A random blood sugar level over 200mg/dcl is an indication of having DM.			
**12.**	Screening of DM at the age of 40 and could be earlier if risk factors existed.			
**13.**	Blood sugar levels management is enough to prevent DM complications.			
**14.**	More women than men are diagnosed with DM			
**15.**	DM is considered one of the main causes of heart diseases.			
**16.**	Soft drinks are a risk factor to developing DM.			

**17.**	Reducing weight is an important way to manage blood sugar level			
**18.**	Foot amputation is mainly caused by DM.			
**19.**	Fundoscopy and retina exam for DM patient is recommended only when it is necessary.			
**20.**	One of DM's main complication is renal failure.			
**21.**	Adapting to DM is important for DM management.			
**No.**	**Physical Activity**			
**1.**	Walking as exercise has health benefits for DM patients			
**2.**	Doing 20-30 minutes of exercise daily is beneficial for people with DM.			
**3.**	Walking 20-30 minutes five times per week can help in preventing DM.			
**4.**	Increasing daily physical activity is important for DM management.			
**5.**	Sedentary behavior such as sitting for long period of time can lead to DM.			
**6.**	Daily physical activity, even it is intermittent for example, for five minutes, can help with DM management.			

Supplementary Table S2: Translated Survey items with results overtime in DM knowledge

DM General Knowledge		2017	2018	2019	2020	P-value (τ)
Obesity causes DM	X	86 (6.8)	71 (7.2)	68 (11.1)	49 (8.4)	0.02 (-0.02)
	✓	1183 (93.2)	918 (92.8)	542 (88.9)	532 (91.6)	
Full or partial shortage of insulin hormone in the blood causes DM	X	228 (18.0)	193 (19.9)	142 (23.3)	124 (21.8)	0.01 (-0.04)
	✓	1040 (82.0)	777 (80.1)	467 (76.7)	444 (78.2)	
Type 1 DM is a chronic disease with no available cure the time being, but it can be managed.	X	324 (25.9)	258 (27.1)	174 (29.0)	182 (32.8)	0.002 (-0.05)
	✓	929 (74.1)	693 (72.9)	425 (71.0)	373 (67.2)	
In healthy individuals, fasting blood sugar levels are considered high if above 100 mg/dcl.	X	711 (57.5)	545 (56.9)	356 (59.7)	359 (64.1)	0.01 (-0.04)
	✓	525 (42.5)	413 (43.1)	240 (40.3)	201 (35.9)	
Eyes functions could be affected by chronic high blood sugar levels and may lead to blindness.	X	190 (15.1)	141 (14.6)	102 (16.9)	107 (18.8)	0.04 (-0.03)
	✓	1067 (84.9)	823 (85.4)	502 (83.1)	462 (81.2)	
Uncontrolled type 1 DM patient should not fast.	X	536 (42.9)	421 (44.0)	245 (40.6)	197 (35.5)	
	✓	712 (57.1)	536 (56.0)	359 (59.4)	358 (64.5)	0.004 (0.05)
Polyuria (frequent urination) during night, sudden/ unexplained weight loss and excessive thirst are symptoms of hyperglycemia.	X	195 (15.4)	144 (14.8)	97 (16.1)	115 (20.2)	
	✓	1072 (84.6)	827 (85.2)	507 (83.9)	455 (79.8)	0.02 (-0.03)
Gestational diabetes mellitus may increase the risk of giving birth to an overweight baby and might lead to her/his death.	X	605 (48.3)	410 (43.2)	288 (47.6)	308 (54.6)	0.03 (-0.03)
	✓	647 (51.7)	540 (56.8)	317 (52.4)	256 (45.4)	
Type 2 DM is a chronic disease with no available cure the time being, but it can be managed.	X	583 (46.9)	405 (42.7)	260 (43.0)	262 (46.8)	0.62 (0.02)
	✓	660 (53.1)	544 (57.3)	344 (57.0)	298 (53.2)	
The prevalence of DM is high in the community and it increased in recent years.	X	144 (11.4)	102 (10.6)	71 (11.7)	79 (13.8)	0.17 (-0.01)
	✓	1117 (88.6)	862 (89.4)	534 (88.3)	492 (86.2)	
A random blood sugar level over 200mg/dcl is an indication of having DM.	X	714 (56.9)	487 (51.3)	334 (55.2)	339 (59.6)	0.39 (-0.01)
	✓	541 (43.1)	463 (48.7)	271 (44.8)	230 (40.4)	
Screening of DM at the age of 40 and could be earlier if risk factors existed.	X	509 (40.5)	400 (41.8)	270 (44.8)	266 (46.7)	0.006 (-0.05)
	✓	749 (59.5)	557 (58.2)	333 (55.2)	304 (53.3)	
Blood sugar levels management is enough to prevent DM complications.	X	323 (25.5)	214 (22.0)	136 (22.3)	166 (28.9)	0.38 (-0.01)
	✓	944 (74.5)	758 (78.0)	473 (77.7)	409 (71.1)	
More women than men are diagnosed with DM.	X	802 (68.0)	581 (63.8)	386 (67.6)	389 (74.1)	0.03 (-0.03)
	✓	377 (32.0)	329 (36.2)	185 (32.4)	136 (25.9)	
	X	607 (51.1)	407 (45.0)	265 (46.9)	277 (52.5)	0.99

DM is considered one of the main causes of heart diseases.	✓	580 (48.9)	498 (55.0)	300 (53.1)	251 (47.5)	(0.01)
Soft drinks are a risk factor to developing DM.	X	247 (20.8)	161 (17.8)	107 (18.9)	93 (17.7)	0.14 (0.02)
	✓	940 (79.2)	742 (82.2)	458 (81.1)	433 (82.3)	
Reducing weight is an important way to manage blood sugar level.	X	150 (12.6)	73 (8.1)	75 (13.3)	67 (12.8)	0.59 (0.00)
	✓	1039 (87.4)	826 (91.9)	488 (86.7)	456 (87.2)	
Foot amputation is mainly caused by DM.	X	313 (26.3)	232 (26.0)	171 (30.1)	181 (34.4)	<0.001 (-0.06)
	✓	878 (73.7)	660 (74.0)	397 (69.9)	345 (65.6)	
Fundoscopy and retina exam for DM patient is recommended only when it is necessary.	X	643 (54.4)	519 (57.8)	283 (49.9)	254 (48.9)	0.01 (0.04)
	✓	540 (45.6)	379 (42.2)	284 (50.1)	265 (51.1)	
One of DM's main complication is renal failure.	X	480 (40.4)	342 (38.0)	229 (40.3)	216 (41.1)	0.77 (0.00)
	✓	708 (59.6)	558 (62.0)	339 (59.7)	310 (58.9)	
Adapting to DM is important for DM management.	X	204 (17.2)	124 (13.7)	70 (12.3)	87 (16.5)	0.24 (0.02)
	✓	985 (82.8)	782 (86.3)	499 (87.7)	440 (83.5)	

Note: τ denotes Kendall's Tau coefficient indicating effect size; ✓ denotes correct answer; X denotes wrong answer; significant at $p<0.05$.

Supplementary Table S3. Translated Survey items with results overtime in Physical Activity

Physical Activity		2017	2018	2019	2020	P-value (τ)
Walking as exercise has health benefits for DM patients	X	58 (4.8)	23 (2.5)	16 (2.8)	17 (3.2)	0.04 (0.02)
	✓	1144 (95.2)	896 (97.5)	557 (97.2)	519 (96.8)	
Doing 20-30 minutes of exercise daily is beneficial for people with DM.	X	95 (7.9)	46 (5.0)	36 (6.3)	34 (6.4)	0.20 (0.015)
	✓	1107 (92.1)	870 (95.0)	538 (93.7)	499 (93.6)	
Walking 20-30 minutes five times per week can help in preventing DM.	X	155 (13.1)	111 (12.3)	78 (13.6)	68 (13.0)	0.89 (0.00)
	✓	1030 (86.9)	790 (87.7)	494 (86.4)	455 (87.0)	
Increasing daily physical activity is important for DM management.	X	198 (16.6)	112 (12.3)	79 (13.9)	68 (12.9)	0.04 (0.03)
	✓	993 (83.4)	798 (87.7)	490 (86.1)	458 (87.1)	
Sedentary behavior such as sitting for long period of time can lead to DM.	X	339 (28.4)	240 (26.5)	149 (26.1)	166 (31.7)	0.42 (0.00)
	✓	853 (71.6)	667 (73.5)	422 (73.9)	358 (68.3)	
Daily physical activity, even it is intermittent for example, for five minutes, can help with DM management.	X	293 (24.6)	208 (22.8)	158 (27.7)	136 (25.7)	0.28 (-0.017)
	✓	900 (75.4)	705 (77.2)	413 (72.3)	393 (74.3)	

Note: τ denotes Kendall's Tau coefficient indicating effect size; ✓ denotes correct answer; X denotes wrong answer; significant at $p<0.05$.
